# Balancing Fertility and Oncologic Uncertainty: Robotic Myomectomy With Bilateral Uterine Artery Ligation for Smooth Muscle Tumor of Uncertain Malignant Potential (STUMP)

**DOI:** 10.7759/cureus.109032

**Published:** 2026-05-17

**Authors:** Serene Mary Saji, Urmila Soman

**Affiliations:** 1 Obstetrics and Gynecology, Aster Medcity, Kochi, IND; 2 Gynecology, Dr. Urmila's Training Centre, Kochi, IND

**Keywords:** fertility preservation, minimally invasive gynecology, robotic myomectomy, stump, uterine artery ligation

## Abstract

Smooth muscle tumor of uncertain malignant potential (STUMP) is a rare uterine neoplasm characterized by ambiguous histopathological features and unpredictable clinical behavior. Management is particularly challenging in young, nulliparous women, where the standard approach of hysterectomy conflicts with fertility preservation. We report a case of a young nulliparous woman who underwent fertility-preserving robotic myomectomy with bilateral uterine artery ligation for a presumed atypical leiomyoma, later diagnosed as STUMP on histopathology. This case highlights the diagnostic limitations of preoperative assessment, the intraoperative decision-making involved in fertility-sparing surgery, and the role of robotic-assisted techniques in optimizing surgical precision and uterine preservation. In the absence of standardized management guidelines, treatment of STUMP requires individualized decision-making, balancing oncologic risk with reproductive goals. Careful patient selection, multidisciplinary evaluation, and long-term surveillance are essential. This case supports the feasibility and safety of a conservative robotic approach in selected patients.

## Introduction

Uterine smooth muscle tumors of uncertain malignant potential (STUMPs) represent a rare and diagnostically challenging subset of uterine neoplasms that lie in the gray zone between benign leiomyomas and malignant leiomyosarcomas. First defined by the World Health Organization in 2003, STUMPs lack definitive histopathologic criteria for benignity or malignancy, making both diagnosis and management inherently complex [1]. Clinically, these tumors often mimic leiomyomas, presenting with abnormal uterine bleeding, pelvic pain, or mass effect, and are frequently diagnosed only after surgical excision [2].

The increasing trend toward delayed childbearing has heightened the importance of fertility-preserving strategies in gynecologic oncology. Myomectomy remains the standard surgical option for women desiring uterine conservation, particularly in symptomatic fibroids [3]. However, when histopathology reveals STUMP, clinicians face a dilemma: while hysterectomy is traditionally recommended due to uncertain malignant potential, it may not be acceptable in young nulliparous women seeking future fertility [4].

Recent literature suggests that fertility-sparing management with myomectomy may be a reasonable alternative in carefully selected patients, with reported pregnancy rates approaching 38-40% and no clear increase in malignant transformation compared to hysterectomy [5]. Nonetheless, recurrence rates remain variable, and long-term oncologic safety is not fully established [5,6].

Advances in minimally invasive surgery, particularly robotic-assisted myomectomy, have further refined surgical outcomes by improving precision, reducing blood loss, and enhancing recovery. Techniques such as bilateral uterine artery ligation or occlusion during myomectomy have been explored to minimize intraoperative hemorrhage, though their impact on ovarian reserve and fertility outcomes remains an area of ongoing research [7,8].

Another critical issue in STUMP management is specimen retrieval. The use of morcellation, especially power morcellation, has been controversial due to the potential risk of disseminating occult malignancy. Contained extraction techniques and careful intraoperative assessment are therefore essential when malignancy cannot be excluded preoperatively [9].

In this context, we present a case of a young nulliparous woman who underwent robotic myomectomy with bilateral uterine artery ligation for a presumed leiomyoma, subsequently diagnosed as STUMP. This case highlights the challenges of preoperative diagnosis, intraoperative decision-making, specimen retrieval, and balancing fertility preservation against oncologic uncertainty. This is a single case study, and further case studies are required to enhance the importance of fertility-sparing myomectomy in cases of doubtful borderline malignancies. This work was previously presented as an oral poster at the All Kerala Congress of Obstetricians and Gynecology 2026 on January 21, 2026.

## Case presentation

**Figure 1:**
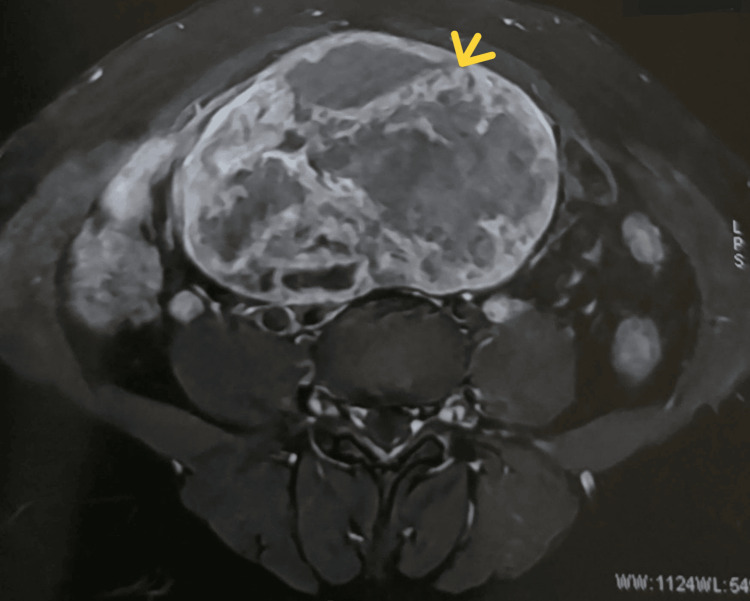
MRI pelvis Axial MR image showing a large 15 x 12 x 11 cm heterogeneous, well-circumscribed uterine mass (yellow arrow), which is irregularly thick, lobulated, heterogeneously enhancing solid lesion with central necrotic cystic areas arising from fundal and posterior wall of uterus

A 25-year-old nulliparous woman, married for one year, presented with complaints of an incidental mass in the abdomen for two weeks and difficulty in voiding. There was no history of abnormal uterine bleeding, dysmenorrhea, weight loss, or constitutional symptoms. Her past medical and surgical history was unremarkable. On clinical examination, the patient was hemodynamically stable. Abdominal examination revealed a palpable, firm, nontender mass arising from the pelvis, corresponding to approximately a 24-week gravid uterus. There was no evidence of ascites or lymphadenopathy. Magnetic resonance imaging (MRI) of the pelvis (Figure 1) demonstrated a large, well-defined uterine mass with heterogeneous signal intensity on T1- and T2-weighted images, showing features suggestive of a degenerative leiomyoma; however, atypical features raised suspicion for STUMP. After detailed counselling regarding the uncertain malignant potential of the lesion, possible need for further surgery, and implications for future fertility, a decision was made to proceed with fertility-preserving surgical management, robotic-assisted myomectomy with bilateral uterine artery ligation.

Surgical procedure

**Figure 2:**
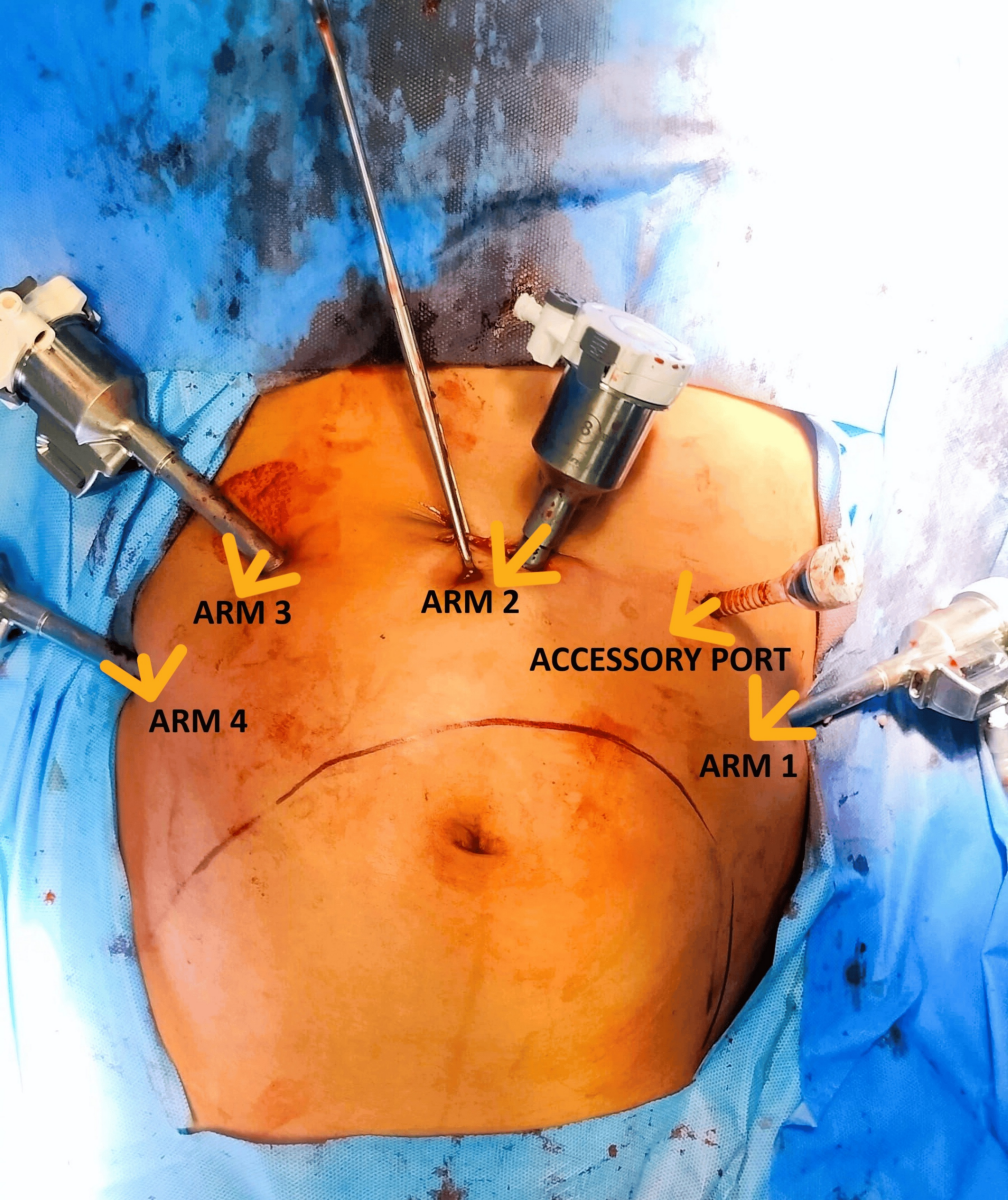
Port placement Robotic ports placed 8 cm apart (arm 1: fenestrated bipolar forceps, followed by 5 mm accessory port; arm 2: endoscope; arm 3: monopolar scissors; arm 4: tenaculum)

The patient underwent robotic-assisted myomectomy with bilateral uterine artery ligation under general anesthesia. The patient was placed in a dorsal steep Trendelenburg position, and widely spaced four robotic ports were put ~8cm apart (Figure 2).

**Figure 3:**
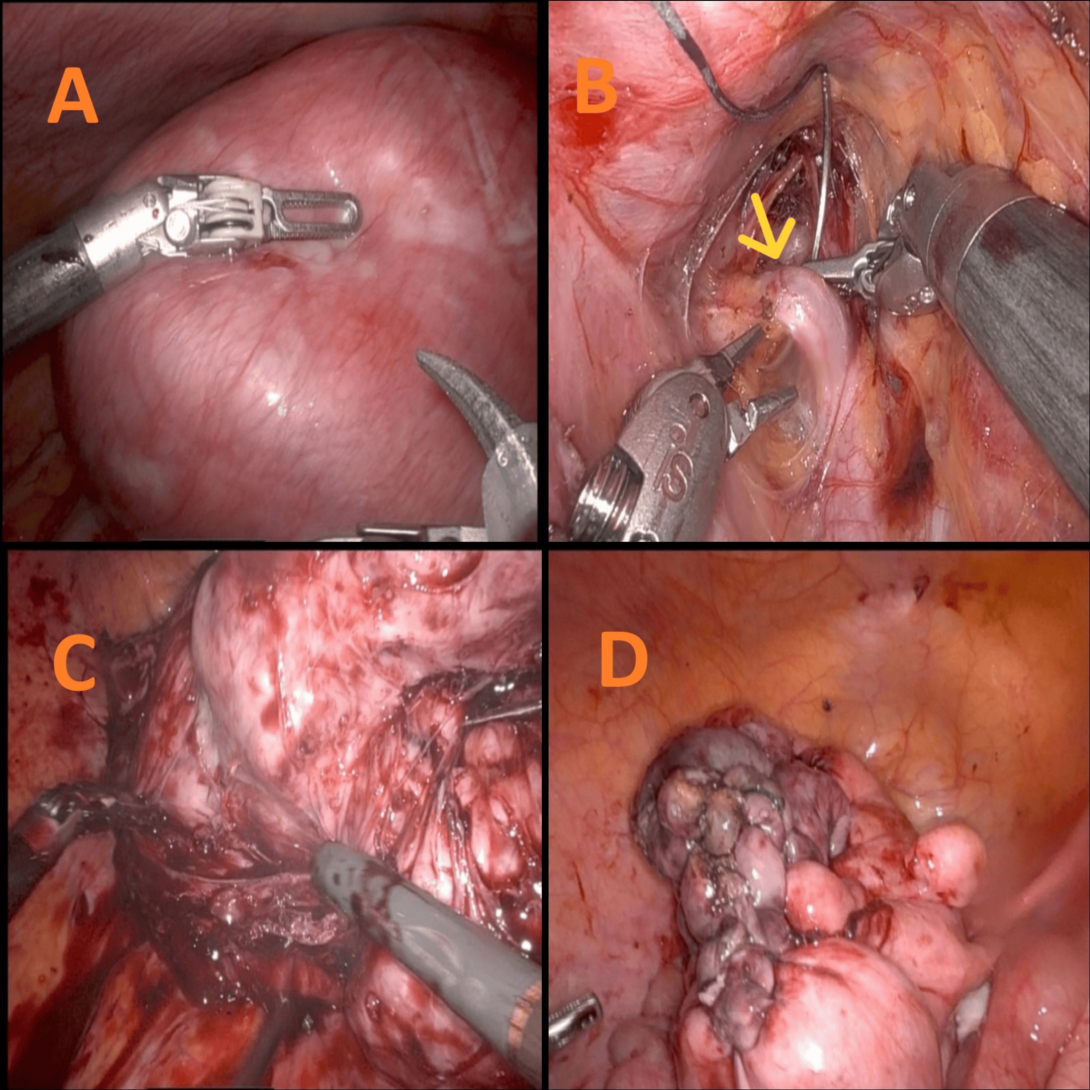
Intraoperative robotic surgical view (A) Vascular degenerative myoma. (B) Uterine artery  (yellow arrow) ligation by a shoelace knot. (C) Myoma enucleation, (D) Repaired myoma bed by flap method

Intraoperatively, a large subserosal fundal myoma measuring approximately 10 × 9 cm was identified, which appeared highly vascular with areas of degeneration (Figure 3A). A total of 40 units of vasopressin was injected into the myoma to reduce intraoperative blood loss. Bilateral uterine artery ligation was performed by posterior approach with careful dissection and skeletonization at the level of the pelvic wall using a shoelace knot technique (Figure 3B). A horizontal incision was made over the myoma, and enucleation was performed meticulously, preserving the surrounding myometrium (Figure 3C). The endometrial cavity was not breached during the procedure, which was confirmed with methylene blue instillation into the endometrial cavity. The specimen was retrieved using contained Endobag morcellation to minimize the risk of tissue dissemination. The myoma bed was repaired with layered suturing by the flap method to restore uterine integrity (Figure 3D). Uterine artery ligatures were released after achieving hemostasis. Bilateral tubes and ovaries were normal. Minimal energy and gentle tissue handling were used to minimize any tissue dissemination. The operative time was 100 minutes, with an estimated blood loss of 350 mL (intraoperative complications: none).

Histopathological examination

**Figure 4:**
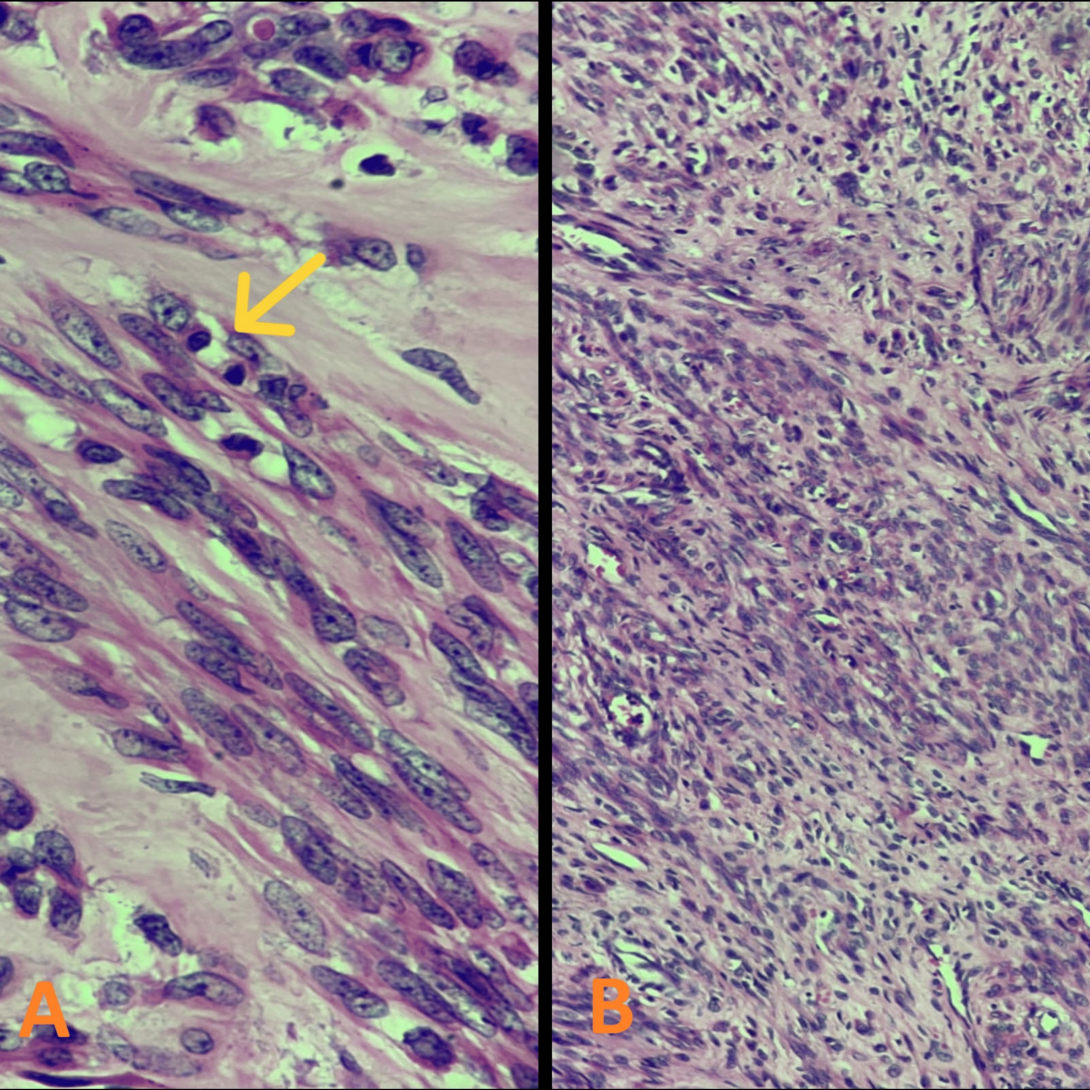
Histopathology (H&E stain, 40x magnification) (A) Cells showing diffuse cellular atypia and increased mitosis (yellow arrow). (B) Section showing cellular neoplasm composed of spindle shaped cells arranged in interlacing fascicles

Gross examination revealed a well-circumscribed mass with a whorled appearance and areas of degeneration. Microscopic examination demonstrated moderate nuclear atypia, low mitotic activity (<10 mitoses per 10 high-power fields), and absence of coagulative tumor cell necrosis (Figures 4A-4B). Surgical margins were free of tumor. These findings were consistent with a diagnosis of STUMP.

Postoperative course and follow-up

The patient had an uneventful postoperative recovery and was discharged on postoperative day one in stable condition. Her case was reviewed in a multidisciplinary tumor board, and a decision was made for conservative management with close surveillance. Follow-up planning included periodic clinical evaluation and imaging studies with pelvic ultrasonography to monitor for recurrence. A three-month follow-up of the patient was uneventful, with the couple trying for conception.

## Discussion

STUMP tumors pose significant challenges due to their unpredictable biological behavior and lack of standardized management guidelines. Their rarity, accounting for approximately 0.01% of uterine smooth muscle tumors, further limits robust evidence-based recommendations [10].

Diagnostic dilemma

Preoperative differentiation between leiomyoma, STUMP, and leiomyosarcoma remains difficult despite advances in imaging. Ultrasound and MRI may suggest atypical features, but definitive diagnosis relies on histopathological evaluation, including assessment of mitotic activity, cytologic atypia, and tumor cell necrosis [2,4]. Consequently, most STUMPs are diagnosed incidentally after myomectomy, as in the present case. This diagnostic uncertainty significantly impacts surgical planning. While conservative surgery may be appropriate for presumed benign fibroids, the unexpected diagnosis of STUMP raises concerns regarding residual disease and recurrence risk.

Fertility Preservation vs Oncologic Safety

The cornerstone of STUMP management has traditionally been hysterectomy, particularly in women who have completed childbearing. However, emerging evidence supports conservative management in young patients desiring fertility. A recent systematic review demonstrated comparable overall survival between patients undergoing myomectomy and hysterectomy, although recurrence rates were slightly higher in the myomectomy group (approximately 11.9% vs 4.1%) [5]. Importantly, pregnancy does not appear to increase recurrence risk, and successful pregnancies following fertility-sparing surgery have been documented [4,6]. Therefore, patient selection becomes critical. Ideal candidates for conservative management include young, nulliparous women with localized disease, absence of high-risk histologic features, and willingness for long-term surveillance.

Role of Robotic Myomectomy and Uterine Artery Ligation

Minimally invasive approaches, including robotic-assisted myomectomy, offer advantages such as enhanced visualization, precise dissection, and reduced perioperative morbidity. In cases with large or vascular fibroids, bilateral uterine artery ligation is an effective adjunct to reduce intraoperative blood loss [11].

However, concerns remain regarding the impact of uterine artery occlusion on ovarian reserve and reproductive outcomes. Current evidence suggests that while pregnancy is possible following uterine artery occlusion combined with myomectomy, further studies are needed to fully evaluate its safety in fertility preservation [7,8]. In the present case, the use of robotic surgery with uterine artery ligation enabled effective tumor removal while minimizing surgical morbidity, an important consideration in young patients.

Specimen Retrieval and Morcellation Concerns

Specimen retrieval represents a critical issue in cases where malignancy cannot be excluded. Power morcellation has been associated with the dissemination of occult sarcoma, leading to worsened prognosis. As a result, contained morcellation techniques or en bloc specimen removal are recommended whenever feasible [9]. In STUMP cases, the risk of dissemination is less clearly defined than in leiomyosarcoma but remains a concern. Therefore, careful intraoperative judgment and adherence to oncologic safety principles are essential.

Follow-up and Surveillance

Given the uncertain malignant potential of STUMP, long-term follow-up is mandatory. Recurrences have been reported even years after initial treatment, underscoring the need for periodic imaging and clinical evaluation [6]. Multidisciplinary management involving gynecologic oncologists, pathologists, and reproductive specialists is crucial to optimize outcomes. Counseling should emphasize the balance between fertility preservation and potential oncologic risks.

## Conclusions

STUMP tumors represent a unique clinical entity characterized by diagnostic ambiguity and unpredictable behavior. Management in young nulliparous women requires careful balancing of fertility preservation with oncologic safety. Robotic myomectomy with bilateral uterine artery ligation can be a feasible and effective fertility-sparing approach in selected patients, offering surgical precision and reduced morbidity. However, the inability to reliably diagnose STUMP preoperatively, combined with concerns regarding specimen retrieval and recurrence, necessitates cautious case selection and thorough counseling. Further studies are necessary to prove the effectiveness of fertility-sparing surgeries with respect to reproductive capabilities. Ultimately, individualized, multidisciplinary decision-making and vigilant long-term follow-up remain the cornerstones of managing STUMP in women desiring future fertility.
